# A cohort study of ethnic differences in use of adjuvant chemotherapy and radiation therapy for breast cancer in New Zealand

**DOI:** 10.1186/s12913-017-2027-4

**Published:** 2017-01-21

**Authors:** Sanjeewa Seneviratne, Ian Campbell, Nina Scott, Ross Lawrenson

**Affiliations:** 1 Waikato Clinical School, University of Auckland, Hamilton, New Zealand; 2 Māori Health Services, Waikato District Health Board, Hamilton, New Zealand; 3 Department of Surgery, University of Colombo, Colombo, Sri Lanka

**Keywords:** Breast cancer, Chemotherapy, Radiation therapy, Ethnicity, Inequity

## Abstract

**Background:**

Ethnic and socioeconomic inequities in use of breast cancer adjuvant therapy are well documented in many countries including the USA, and are known to contribute to lower breast cancer survival among minority ethnic and socioeconomically deprived women. We investigated ethnic and socioeconomic inequities in use of adjuvant radiotherapy and chemotherapy in a cohort of women with invasive breast cancer in New Zealand.

**Methods:**

All women with newly diagnosed invasive breast cancer during 1999-2012 were identified from the Waikato Breast Cancer Register. Rates of chemotherapy use and radiotherapy use were assessed in women who were deemed to be eligible for chemotherapy (*n* = 1212) and radiotherapy (*n* = 1708) based on guidelines. Factors associated with use of chemotherapy and radiation therapy were analysed in univariate and multivariate regression models, adjusting for covariates.

**Results:**

Overall, rates of chemotherapy and radiotherapy use were 69% (*n* = 836) and 87.3% (*n* = 1491), respectively. In the multivariate model, significantly lower rates of radiotherapy use were associated with Māori compared with NZ European (Odds Ratio [OR] = 0.63, 0.40-0.98), presence of comorbidity (OR = 0.49, 0.34-0.72), distance from hospital of over 100km (OR = 0.47, 0.23-0.96), mastectomy compared with breast conserving surgery (OR = 0.32, 0.17-0.56) and non-screen compared with screen detection (OR = 0.53, 0.35-0.79). No significant associations were observed between chemotherapy use and ethnic or socio-demographic factors.

**Conclusions:**

Improving access for radiotherapy, especially for women who are at a higher risk of not receiving optimum cancer therapy due to ethnicity, geography or socioeconomic status need to be recognized as a priority to reduce inequities in breast cancer care in New Zealand.

## Background

Disparities in breast cancer survival by ethnicity and socioeconomic status are well documented in many countries [1–4]. As in the USA, poor healthcare access among ethnic minority or Indigenous and socioeconomically deprived women has been shown to be a major factor for such breast cancer survival disparities [2]. Differences in quality and timeliness in treatment of breast cancer, including differences in the use of adjuvant therapy have also been reported to be important contributors for ethnic and socioeconomic disparities in breast cancer survival [1, 5, 6].

Indigenous Māori in New Zealand are known to have lower access, receive inferior quality cancer care and experience longer cancer treatment delays compared with non-Indigenous NZ Europeans for a variety of cancers [7–10]. For instance, Māori patients have been reported to experience longer delays for surgical treatment of breast and lung cancer, and to have a lower likelihood of receiving chemotherapy for bowel cancer compared with NZ European patients [7, 8]. Breast cancer mortality rate in Māori is 60% higher compared with NZ European women, and more advanced cancer stage at diagnosis in Māori has been shown to be the major factor towards this disparity [11]. However, data are sparse on possible ethnic differences in use, quality or timeliness of adjuvant therapy for breast cancer in New Zealand.

New Zealanders receive healthcare through a mixture of public and privately-funded services. Publicly funded health service is well resourced and provides free specialist and hospital care to all citizens. Private health care facilities run parallel to the public and are mostly funded through insurance schemes. BreastScreen Aotearoa (BSA) is the national breast cancer screening programme which provides free biennial breast cancer screening for all women aged 45-69 years. The Waikato District Health Board region has a population of just over 400,000. It has a major urban centre, a significant rural population and a Māori population of nearly 84,000 [12]. While a majority of women receive surgical care through the public sector a minority receive surgical care through well-equipped private sector hospitals. Oncology services for the region are available only through the public sector. Radiation facilities for the region are provided exclusively through the radiation facility at the tertiary hospital in Hamilton. A majority receive chemotherapy through the same tertiary centre in Hamilton while a minority receive chemotherapy through a satellite facility.

We hypothesized that Māori women were less likely to have received recommended adjuvant chemotherapy and/or radiotherapy compared with NZ European women [13, 14], which might have contributed to the higher breast cancer mortality in Māori women. To answer this question, we analysed cancer treatment data from a regional, population based sample of women with newly diagnosed breast cancer over a period of 14 years. Rates of adjuvant chemotherapy and radiotherapy use by socio-demographic and tumour characteristics were analysed individually, and adjusting for covariates, to identify associations between use of adjuvant therapy, and socio-demographic characteristics.

## Methods

### Study population

Data for this study were extracted from the Waikato Breast Cancer Register (WBCR), a prospective database of newly diagnosed breast cancers in the Waikato, New Zealand since 1999. Completeness and accuracy of the WBCR data have been validated previously [15]. All women with newly diagnosed primary invasive breast cancer during the period from 01/01/1999 through 31/12/2012, were identified from the WBCR (*n* = 2848). Of this, women with metastatic cancer at diagnosis (*n* = 166) and women who did not undergo primary surgery (*n* = 114) were excluded.

### Data

Patient ethnicity was obtained from the WBCR which records self-assigned ethnicity and was grouped into four categories; Māori, Pacific, NZ European, and Other. Cancer staging was performed according to TNM (Tumour, Lymph node and Metastasis) staging system [16].

Socioeconomic status was categorized according to New Zealand Deprivation Index 2006 (NZDep06) [17]. NZDep06 measures socioeconomic status based on area of residence and assigns a deprivation score on a scale from 1 to 10 (1-least deprived 10% of areas, 10-most deprived 10% of areas in NZ) based on nine socioeconomic parameters. Distance to treatment facility where surgery was carried out was calculated based on patients’ residential address and was categorized in to four categories; 0-10km, 10-50km, 50-100km and >100km. A comorbidity score for each woman was calculated using Charlson Comorbidity Index [18], based on existing comorbidities at the time of diagnosis of breast cancer. Comorbidity score was categorized into 0 or ≥1.

### Use of adjuvant therapy

#### Chemotherapy

**Fig. 1:**
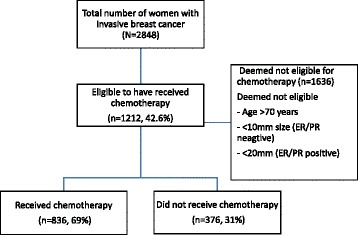
Flow diagram demonstrating the eligibility criteria and number of women who have received chemotherapy

Chemotherapy eligibility was considered only for women younger than 70 years. Of the women considered to be eligible for chemotherapy (*N* = 1212), women who received either adjuvant or neo-adjuvant chemotherapy were considered to have received chemotherapy. For oestrogen (ER) and progesterone (PR) receptor negative cancers, a maximum tumour diameter of ≥10mm (*n* = 276, 22.8%) was considered as the threshold for chemotherapy (Fig. 1). For ER and/or PR positive tumours, defining a threshold for chemotherapy was complicated as this decision in most situations was based on multiple factors including lymph node involvement, tumour grade, lympho-vascular invasion, human epidermal growth factor receptor – type 2 (HER-2) status, and more recently, with Ki-67 and tumour genotyping [19]. For ER and/or PR positive or unknown tumours, we considered ≥20mm maximum tumour diameter as the threshold for chemotherapy (*n* = 936, 77.2%) [13, 14]. We also performed a separate analysis with a different threshold for ER and/or PR positive cancers. For this analysis, women were considered eligible only if one or more of lymph node positivity, tumour grade ≥2 or lympho-vascular invasion were present, in addition to a maximum tumour diameter of ≥20mm.

#### Radiotherapy

**Fig. 2:**
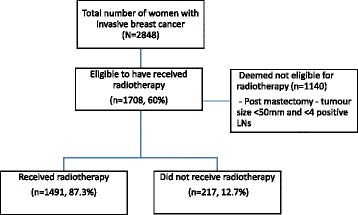
Flow diagram demonstrating the eligibility criteria and number of women who have received radiotherapy

Women who were deemed to be eligible for radiotherapy (*n* = 1708) were identified based on following criteria (Fig. 2). All women undergoing breast conserving surgery without a completion mastectomy (*n* = 1354, 79.3%) were considered eligible, and for women undergoing a mastectomy, if the maximum tumour diameter was ≥50mm or if ≥4 lymph nodes were positive for tumour metastasis (*n* = 354, 20.7%) were considered eligible [13, 14].

### Data analysis

Data were analysed using SPSS (Version 22) [20]. Chi squared tests (χ^2^) for trend or Wilcoxson rank test were used to test differences in use of chemotherapy and radiotherapy among groups categorized by age, ethnicity, stage, mode of diagnosis and year of diagnosis. Multivariable logistic regression was used to derive relative odds (odds ratios) of Maori women receiving chemotherapy or radiotherapy compared with NZ European ethnicity adjusted for other factors. Variables were retained in multivariable models if p values were less than, or equal to the conventional 5% level, or if they were considered to be of significant clinical or population health importance. Possible interactions between covariates included in multivariate models were not studied.

Ethnic comparisons were performed between NZ European (*n* = 2303, 80.9%) and Maori (*n* = 429, 15.1%) populations. Pacific (*n* = 53, 1.9%) and Asian (*n* = 63, 2.2%) populations were excluded from comparisons due to small sample size.

Logistic regression analyses were performed including only the patients with complete data for all variables of interest. Patients with missing data were excluded from regression models as the missing numbers were relatively small (<5%), except for HER-2 status (missing data 23.7%). Regression analyses for chemotherapy was performed twice; firstly, including missing HER-2 status as a separate category and secondly, excluding cases with missing HER-2 status data. Odds ratios and p values between the two models were found to be almost identical. The model with missing data as a separate category is shown in this report. Imputation of missing values was not undertaken due to the similarity of these results.

## Results

### Use of chemotherapy

Of women deemed eligible for chemotherapy, 836 (69%) women had received chemotherapy. No significant differences in rates of chemotherapy use were observed between Māori and NZ European women (68.3% vs. 68.7%, *p* = 0.916). Chemotherapy use was significantly higher in women of younger age groups (*p* < 0.001), zero comorbidity score (*p* < 0.001), surgically treated in private hospitals (*p* = 0.002) and non-screen detected cancer (*p* = 0.033) (Table 1). Increasing socioeconomic deprivation tended to be associated with a lower use of chemotherapy, although this was not statistically significant (*p* = 0.402). As expected, chemotherapy use was higher for cancers which were associated with adverse prognostic characteristics, which included ≥5cm in diameter, positive lymph node status, higher grade, lympho-vascular invasion (LVI) and HER-2 positivity. Trends in use of chemotherapy by tumour characteristics were observed to be similar for Māori and NZ European women. Among these women deemed eligible for chemotherapy, no significant differences in the distribution of tumour biological characteristics between Maori and NZ European women were observed (Data not shown).

**Table 1:** Socio-demographic and tumour characteristics associated with use of adjuvant chemotherapy^a^

		Use of chemotherapy			
Characteristic	Total population (*N* = 1212)	Total chemotherapy (*N* = 1212)		NZ European (*N* = 924)	Māori (*N* = 218)
	*n* (%)	*n* (%)^b^	*p*	*n* (%)^b^	*n* (%)^b^
Overall	1212 (100)	836 (69.0)		635 (68.7)	149 (68.3)
Age (yrs.)			<0.001		
< 40	100 (8.3)	90 (90.0)		58 (89.2)	18 (90.0)
40-49	338 (27.9)	276 (81.7)		200 (81.3)	56 (81.2)
50-59	434 (35.8)	309 (71.2)		245 (72.5)	53 (68.8)
60-69	340 (28.1)	161 (47.4)		132 (48.0)	22 (42.3)
Deprivation			0.402		
Dep 1-2	139 (11.5)	100 (71.9)		88 (71.5)	8 (80.0)
Dep 3-4	126 (10.4)	89 (70.6)		72 (70.6)	12 (75.0)
Dep 5-6	313 (25.8)	216 (69.0)		174 (67.7)	29 (70.7)
Dep 7-8	315 (26.0)	213 (67.6)		160 (67.8)	39 (61.9)
Dep 9-10	319 (26.3)	218 (68.3)		141 (68.4)	61 (69.3)
Surgical hospital type			0.002		
Private	406 (33.5)	303 (74.6)		271 (74.0)	21 (80.8)
Public	806 (66.5)	533 (66.1)		364 (65.2)	128 (66.7)
Diagnostic type			0.033		
Screen detected	407 (33.6)	235 (57.7)		195 (57.9)	30 (57.7)
Non-screen detected	805 (66.4)	601 (74.7)		440 (75.0)	119 (71.7)
Charlson score			<0.001		
0	1056 (87.1)	754 (71.4)		582 (70.5)	124 (71.7)
1+	156 (12.9)	82 (52.6)		53 (53.5)	25 (55.6)
Diagnosis year			0.003		
1999-2002	269 (22.2)	201 (74.7)		160 (73.7)	31 (79.5)
2003-2006	374 (30.9)	267 (71.4)		216 (72.5)	38 (66.7)
2007-2009	292 (24.1)	183 (62.7)		128 (60.7)	39 (65.0)
2010-2012	277 (22.9)	185 (66.8)		131 (66.2)	41 (66.1)
Grade			<0.001		
Grade I	168 (13.9)	70 (41.7)		62 (43.4)	6 (33.3)
Grade II	617 (50.9)	409 (66.3)		305 (66.0)	83 (66.9)
Grade III	395 (32.6)	340 (86.1)		254 (85.8)	57 (83.8)
Missing	32 (2.6)	17 (53.1)		14 (60.9)	3 (37.5)
ER/PR status			<0.001		
ER &/or PR +	926 (76.4)	598 (64.6)		457 (64.3)	103 (63.6)
ER & PR -	276 (22.8)	233 (84.4)		173 (84.4)	46 (83.6)
Missing	10 (0.8)	5 (50.0)		5 (62.5)	0
T stage			<0.001		
T1	368 (30.4)	234 (63.6)		196 (62,6)	29 (69.0)
T2	692 (57.1)	484 (69.9)		364 (71.4)	83 (62.4)
T3	83 (6.8)	64 (77.1)		40 (74.1)	19 (82.6)
T4	62 (5.1)	50 (80.6)		31 (77.5)	18 (90.0)
Missing	7 (0.6)	4 (55.6)		4 (55.6)	0
N stage			<0.001		
0	406 (33.5)	223 (54.9)		165 (54.6)	37 (50.0)
1	537 (44.3)	381 (70.9)		291 (70.0)	72 (74.2)
2+	269 (22.2)	232 (86.2)		179 (86.9)	40 (85.1)
LVI			<0.001		
Negative	783 (64.6)	484 (61.8)		362 (61.0)	88 (62.0)
Positive	429 (35.4)	352 (82.1)		273 (82.5)	61 (80.3)
HER-2			<0.001		
Negative	658 (54.3)	414 (62.9)		314 (63.2)	79 (60.8)
Equivocal	48 (4.0)	25 (52.1)		17 (48.6)	6 (66.7)
Positive	219 (18.1)	188 (85.8)		134 (86.5)	37 (80.4)
Missing	287 (23.7)	209 (72.8)		170 (71.7)	27 (81.8)

^a^Only ER and PR negative cancers ≥10mm and ER and/or PR positive cancers ≥20mm in women <70 years are included

^b^Proportion of women who had received chemotherapy in each category

Multivariate analysis of factors associated with chemotherapy use is shown in Table 2. Age, comorbidity score and adverse tumour characteristics remained significant while socio-demographic factors and surgical hospital type were not significant.

**Table 2:** Multivariable logistic regression analysis for factors associated with use of adjuvant chemotherapy (Number of patients included in the regression analysis = 1064 [87.8%])

Characteristic	OR	95% CI	*p*
Māori ethnicity^a^	1.02	0.90-1.14	0.934
Age^b^	0.92	0.91-0.94	<0.001
Year of diagnosis^c^	0.94	0.88-1.02	0.122
ER and/or PR positive^d^	0.34	0.21-0.57	<0.001
Deprivation^e^	0.98	0.87-1.11	0.733
Charlson score	0.34	0.17-0.60	<0.001
Surgery in public vs. private	1.07	0.77-1.49	0.688
T stage^f^			
T 2	1.58	1.12-2.23	0.009
T 3	1.47	0.65-2.49	0.486
T 4	1.23	0.41-3.41	0.876
N stage^g^			
N 1	2.49	1.71-3.64	<0.001
N 2+	8.41	4.37-16.2	<0.001
Grade^h^			
Grade II	2.41	1.58-3.70	<0.001
Grade III	4.79	2.87-7.99	<0.001
LVI^i^	1.78	1.22-2.61	<0.001
HER-2^j^			
HER-2 Equivocal	0.55	0.26-1.15	0.116
HER-2 Positive	2.02	1.23-3.33	0.006
HER-2 Unknown	1.36	0.83-2.24	0.548

^a^Maori compared with NZ European ethnicity

^b^Age as a continuous variable

^c^Year of diagnosis as a continuous variable

^d^ER and/or PR positive compared with ER & PR negative

^e^Deprivation as a continuous variable

^f^Reference category T1 stage

^g^Reference category N0 stage

^h^Reference category Grade I

^i^Reference category LVI negative

^j^Reference category HER-2 negative

An additional analysis was performed with a different chemotherapy threshold for ER and/or PR positive cancers, considering these cancers as eligible for chemotherapy only if the cancer had one or more of lymph node positivity, lympho-vascular invasion or tumour grade ≥2 in addition to a maximum tumour diameter of ≥20mm. This analysis yielded results much similar to the analysis in Table 1 (data not shown). According to new criteria, 1168 women were found to be eligible, and of this 824 (70.5%) had received chemotherapy; 623 (70.1%) of NZ European and 149 (70.3%) of Māori women. Multivariable logistic regression analysis showed trends similar to Table 2 and, for Māori, the adjusted odds for receiving chemotherapy was 1.25 (0.85-1.87, *p* = 0.258).

### Use of radiotherapy

Characteristics associated with use of radiotherapy are shown in Table 3. Overall, radiotherapy was used for 1491 (87.3%) of women deemed to be eligible for radiation based on selection criteria. Radiotherapy use was lower in Māori compared with NZ European women (84% vs. 87.8%, *p* = 0.138), but the difference was statistically not significant. Younger age at diagnosis, lower socioeconomic deprivation, later year of diagnosis, surgical care in a private hospital, shorter distance from the hospital, screen detection, undergoing BCS and adverse tumour characteristics including higher grade, stage and positive axillary lymph node status were significantly associated with increased likelihoods of receiving radiotherapy.

**Table 3:** Socio-demographic and tumour characteristics associated with use of adjuvant radiotherapy

		Use of radiotherapy			
Characteristic	Total population (*N* = 1708)	Total (*N* = 1708)		NZ European (*N* = 1418)	Māori (*N* = 225)
	*n* (%)	*n* (%)^a^	*p*	*n* (%)^a^	*n* (%)^a^
Overall	1708	1491 (87.3)		1255 (87.8)	189 (84.0)
Age (yrs.)			<0.001		
< 40	79 (4.6)	76 (96.2)		50 (96.2)	16 (94.1)
40-49	328 (19.2)	302 (92.1)		231 (93.9)	52 (86.7)
50-59	499 (29.2)	456 (91.4)		377 (92.0)	66 (90.4)
60-69	471 (27.6)	417 (88.5)		361 (90.5)	45 (75.0)
70-79	218 (12.8)	172 (78.9)		161 (78.9)	8 (72.7)
80+	113 (6.6)	68 (60.2)		65 (60.7)	2 (50.0)
Diagnosis year			0.003		
1999-2002	357 (20.9)	296 (82.9)		255 (83.9)	30 (76.9)
2003-2006	506 (29.6)	443 (87.5)		396 (88.0)	37 (86.0)
2007-2009	406 (23.8)	354 (87.2)		284 (87.4)	50 (84.7)
2010-2012	439 (25.7)	398 (90.7)		310 (91.4)	72 (85.7)
Deprivation			0.006		
Dep 1-2	178 (10.4)	167 (93.8)		156 (94.0)	5 (83.3)
Dep 3-4	186 (10.9)	163 (87.6)		140 (87.5)	17 (94.4)
Dep 5-6	414 (24.2)	364 (87.9)		318 (88.1)	37 (90.2)
Dep 7-8	491 (28.7)	423 (86.2)		356 (87.9)	54 (77.1)
Dep 9-10	439 (25.7)	374 (85.2)		275 (84.4)	76 (84.4)
Distance			0.005		
< 10km	546 (32.0)	489 (89.6)		409 (90.5)	54 (85.7)
10-50km	660 (38.6)	579 (89.1)		488 (88.1)	70 (85.6)
50-100km	428 (25.1)	364 (85.0)		310 (85.4)	49 (83.1)
> 100km	74 (4.3)	59 (79.7)		38 (79.2)	16 (76.2)
Missing					
Diagnostic type			<0.001		
Screen detected	750 (43.9)	698 (93.1)		602 (93.3)	76 (90.5)
Symptomatic	958 (56.1)	793 (82.8)		643 (83.2)	113 (80.1)
Hospital type			<0.001		
Private	535 (31.3)	488 (91.2)		453 (91.5)	25 (86.2)
Public	1173 (68.7)	1003 (85.5)		792 (85.8)	164 (83.7)
Surgery type			<0.001		
BCS	1354 (79.3)	1213 (89.6)		1031 (89.7)	143 (88.8)
Mastectomy	354 (20.7)	278 (78.5)		214 (79.9)	46 (71.9)
Grade			0.493		
Grade I	441 (25.8)	381 (86.4)		341 (87.0)	27 (79.4)
Grade II	865 (50.6)	754 (87.2)		626 (88.0)	102 (82.9)
Grade III	371 (21.7)	331 (89.2)		257 (89.2)	56 (87.5)
Unknown	31 (1.8)	25 (80.6)		21 (77.8)	4 (100)
T stage			<0.001		
T1	1020 (59.7)	915 (90.1)		790 (90.1)	98 (89.9)
T2	518 (30.3)	437 (84.4)		356 (85.8)	57 (76.0)
T3	100 (5.9)	79 (79.0)		54 (76.1)	20 (87.0)
T4	70 (4.1)	58 (82.9)		43 (84.3)	14 (77.8)
N stage			0.042		
0	1029 (60.3)	900 (87.8)		762 (87.8)	106 (86.9)
1	363 (21.2)	321 (88.9)		266 (90.2)	45 (83.3)
2+	316 (18.5)	266 (84.2)		215 (93.1)	36 (76.6)
Charlson score			<0.001		
0	1456 (85.2)	1312 (90.1)		1111 (90.7)	151 (85.8)
1+	252 (14.8)	179 (71.0)		134 (69.4)	38 (77.6)

^a^Proportion of women who had received radiotherapy in each category

Multivariable regression analysis of factors associated with radiotherapy use is shown in Table 4. Māori compared with NZ European ethnicity (OR = 0.63, 95% CI, 0.40-0.98), older age (OR = 0.96, 95% CI 0.95-0.98), distance of over 100km from the radiation facility (OR = 0.47, 95% CI, 0.23-0.96, higher comorbidity score (OR = 0.49, 95% CI, 0.34-0.72), mastectomy compared with BCS (OR = 0.32, 95% CI, 0.17-0.57) and non-screen compared with screen detection (OR = 0.53, 95% CI, 0.35-0.79) were significantly associated with lower likelihoods of receiving radiotherapy in this model.

**Table 4:** Multivariable logistic regression analysis for factors associated with use of adjuvant radiotherapy (Number of patients included in the regression analysis = 1643 [96.2%])

Characteristic	OR	95% CI	*p*
Māori ethnicity^a^	0.63	0.40-0.98	0.040
Age^b^	0.96	0.95-0.98	<0.001
Year of diagnosis^c^	1.07	1.02-1.11	0.004
Deprivation^d^	0.92	0.79-1.06	0.232
Distance^e^			
10-50 km	0.74	0.50-1.09	0.130
50-100 km	0.67	0.44-1.03	0.067
> 100km	0.47	0.23-0.96	0.040
Charlson score^f^	0.49	0.34-0.72	<0.001
Surgery in public vs. private	0.86	0.61-1.26	0.467
Non-screen vs. screen detection	0.53	0.35-0.79	0.002
Mastectomy vs. BCS	0.32	0.17-0.57	<0.001
T stage^g^			
T 2	0.84	0.57-1.24	0.394
T 3	1.09	0.53-2.24	0.809
T 4	1.42	0.66-3.07	0.366
N stage^h^			
N 1	1.45	0.94-2.24	0.093
N 2+	2.66	1.38-5.10	0.003

^a^Maori compared with NZ European ethnicity

^b^Age as a continuous variable

^c^Year of diagnosis as a continuous variable

^d^Deprivation as a continuous variable

^e^Reference category Distance <10km

^f^Reference category Charlson score = 0

^g^Reference category T1 stage

^h^Reference category N0 stage

Further analyses were performed for women undergoing BCS and mastectomy separately (Data not shown). These analyses confirmed that Māori were less likely to have received radiation following mastectomy (OR = 0.54, 0.24-1.22, *p* = 0.134) and BCS (OR = 0.70, 0.41-1.43, *p* = 0.402), although these differences were not statistically significant. Significantly lower likelihoods of receiving radiation following both BCS and mastectomy were seen for women of older age (OR = 0.94, 95% CI 0.92-0.96 and OR = 0.95, 95% CI 0.93-0.97 respectively), non-screen compared with screen detected (OR = 0.43, 95% CI, 0.31-0.55 and OR = 0.72, 95% CI 0.31-1.70, respectively) and for women with comorbidity (OR = 0.32, 95% CI 0.21-0.43 and OR = 0.28, 95% CI 0.16-0.52, respectively).

## Discussion

This study has shown that use of adjuvant radiotherapy has been significantly lower in Indigenous Māori compared with NZ European women with breast cancer, based on accepted practice guidelines over the study period [13, 14]. No significant difference in the use of chemotherapy was observed between Māori and NZ European women. Further, significantly lower use of radiotherapy was seen among rural compared with urban dwelling women and non-screen compared with screen detected women. Overall, the use of radiation was lower than expected based on guidelines [13, 14], and was substantially worse for post-mastectomy radiation (78.5%) than for radiation following BCS (89.6%). Although the use of radiotherapy seems to have increased over time, a substantial proportion of potentially eligible women (9%) have not received radiation even during 2010-2012.

Lower use of adjuvant chemotherapy in minority ethnic cancer patients are well documented in the USA, and include lower use of chemotherapy for breast, colon and lung among many other cancers [21, 22]. Not only that these patients have experienced lower use of adjuvant chemotherapy, but on many occasions were subjected to longer delays and use of chemotherapy regimens not in keeping with recommended guidelines [23–25]. Similarly, lower use and longer delays for adjuvant chemotherapy for bowel cancer in Māori compared with non-Māori patients have supported the existence of similar ethnic disparities in New Zealand [7]. Despite that, we did not observe a significant difference between Māori and NZ European women in the use of adjuvant chemotherapy for breast cancer, either in univariate or multivariate models. However, a previous analysis based on the WBCR found that Māori women were significantly more likely to experience longer delays for initiation of chemotherapy than for NZ European women [9]. Further, we have not analysed the use of recommended regimens of chemotherapy or rates of completion of chemotherapy in the present study. Hence, although we have not observed an ethnic disparity in overall adjuvant chemotherapy use, further research is needed to investigate possible disparities in other areas of chemotherapy use including rates of completion and use of recommended regimens.

Overall, use of radiotherapy fell short of recommended guidelines, and was significantly lower for Māori compared with NZ European women [13, 14]. Similar inequities in the use of adjuvant radiotherapy for breast cancer have been reported from the USA, between minority African American and White American women [21, 26]. It appears that socio-demographically disadvantaged women (i.e. Māori, rural residence and high socioeconomic deprivation) had higher likelihoods of not receiving adjuvant radiation, while no such differences were observed for chemotherapy. Differences in difficulty in accessing radiotherapy in comparison to chemotherapy might have at least partially been responsible for this difference. Adjuvant radiation for the study population was provided through the central radiation facility at the tertiary hospital in Hamilton. As radiotherapy requires attending a radiation facility five days a week over a period, ranging from four to six weeks, for women residing in remote and rural areas this would have posed a significant barrier due to difficulties with time and cost of travel. Many rural women with breast cancers suitable for BCS opting for mastectomy due to similar reasons is well documented in the literature [27]. Women of low socioeconomic groups also face similar barriers due to difficulties with transport, taking time off work or due to lack of support to care for dependants, resulting in lower use of radiotherapy [28]. Higher proportions of Māori live in rural areas and are more likely to be socioeconomically deprived contributing to lower radiotherapy use in Māori. However, Maori ethnicity appears to be an independent risk factor for lower use of radiotherapy as observed in the multivariate model.

Women with screen detected cancer were significantly more likely to have received radiotherapy compared to women with non-screen detected women, a common pattern following both mastectomy and BCS. Diagnostic and treatment indicators for women diagnosed through BSA programme are routinely measured and performance of each screening provider is regularly audited against pre-established criteria. For instance, at least 95% of women diagnosed through BSA are expected to have received radiotherapy following BCS for invasive cancer [29]. If a provider fails to achieve these targets corrective measures are initiated through a feedback process. However, similar quality measures or audit processes were non-existent for symptomatically detected cancer. This provides a likely explanation for higher radiotherapy rates seen for screen detected cancer, despite these cancers generally carrying a lower risk of local recurrence compared with non-screen detected cancer. This observation highlights a failure of the healthcare system, where women with lower risk cancers have likely been prioritized to receive treatment over women with higher risk cancers. Such inequities in care are likely to further exacerbate inequities in breast cancer outcomes seen between Māori and NZ European women, especially since Māori women have a significantly lower screening coverage [29], and as a result, a lower proportion of screen detected cancer.

Main strengths of this study include the completeness of the population based sample which included more than 98% of all breast cancers diagnosed in the Waikato region over the study period and comprehensive nature of the data included [15]. As the population distributions, provision of breast screening and treatment services in the Waikato region are much similar to rest of New Zealand, and hence findings from this study are likely to be representative of the whole country [12, 29, 30].

However, there were several limitations. First, although we observed differences in adjuvant therapy among some groups of interest and several associations, we could not ascertain exact causes for non-use (i.e. not referred, not seen by an oncologist or patient declined) due to non-availability of this information from the WBCR. Selection of patients for chemotherapy is complicated and is based on multiple factors including age, tumour size, grade, ER/PR, lymph node status and lympho-vascular invasion. As a result, criteria used for eligibility for chemotherapy were not absolute, especially for women with ER/PR positive cancers. This is a likely reason for the much lower use of chemotherapy use in the selected population (69%) compared with radiotherapy use (87.3%), for which the eligibility criteria were less complicated. Further, no major differences in NZ treatment guidelines for the use of breast cancer adjuvant therapy was observed during the study period [13]. Hence, any impact of such changes in guidelines are unlikely to have influenced the study findings. As we did not observe major differences in distribution of tumour biological characteristics between Māori and NZ European women that might have influenced the use of chemotherapy, such factors are unlikely to have influenced patient selection for chemotherapy in a differential manner.

## Conclusion

In conclusion, we observed significantly lower use of radiotherapy for Māori and women living at a distance of >100km from the hospital, although similar disparities were not observed for chemotherapy. Difficulties in accessing radiotherapy appeared to be a major contributor towards differences observed by ethnicity, geographic location and socioeconomic status. Failures of the healthcare system to providing equitable care were also evident by the discrepancy in radiotherapy seen between screen and non-screen detected women. Increasing availability and improving access for breast cancer adjuvant therapy for women who are at a higher risk of not receiving adjuvant therapy due to ethnicity, geography or socioeconomic position need to be recognized as priorities, which may help minimize breast cancer outcome inequities.

## Abbreviations

BCS: Breast conserving surgery
BSA: BreastScreen Aotearoa
ER: Oestrogen receptor
HER 2: Human epidermal growth factor receptor type 2
NZ: New Zealand
NZDep06: New Zealand deprivation index 2006
OR: Odds ratio
PR: Progesterone receptor
TNM: Tumour, Node, Metastasis
WBCR: Waikato breast cancer register
